# Scalability properties of multimodular networks with dynamic gating

**DOI:** 10.1186/1471-2202-14-S1-P136

**Published:** 2013-07-08

**Authors:** Daniel Martí, Omri Barak, Mattia Rigotti, Stefano Fusi

**Affiliations:** 1 Center for Theoretical Neuroscience, Columbia University, New York, NY 10032, USA; 2 Group for Neural Theory, École Normale Supérieure, Paris 75005, France; 3 Technion, Israel Institute of Technology, Haifa, Israel; 4 Center for Neural Science, New York University, New York, NY 10003, USA

## 

Brain processes arise from the interaction of a vast number of neurons. While the number of participating elements is enormous, interactions are generally limited by physical and metabolic constraints---a neuron is connected to thousands other neurons, a far lower number than the hundred billion neurons in the brain. Unfortunately, it is the number of connections per neuron, and not the total number of neurons, what often determines the performance of large neural networks (measured, e.g., as memory capacity), a fact that hinders the scalability of these systems.

We hypothesize that the scalability problem can be circumvented by using multimodular architectures, in which individual modules composed of local, densely connected recurrent networks interact with one another through sparse connections. We propose a general model of multimodular attractor neural networks in which each module state changes only upon external event and the change depends on the state of a few other modules. To implement this scheme, every module has to disregard the state of any module not involved in a particular interaction. Because a module can potentially interact with several others, ignoring the states of non-relevant modules would require learning of an exponentially large number of conditions.

We solve this problem by adding a group of neurons that dynamically gate the interactions between modules. These neurons receive inputs from the modules and event signals through random sparse connections, and respond to combinations of event-states. This information is then sent back to the modules. Because they implement conjunctive representations, the number of necessary gating neurons grows only polynomially with the number of modules. We hypothesize that gating neurons reside in cortical layer 2/3, and that they mediate the interactions between modules in layer 5/6. The laminar organization of the neocortex could thus be a crucial architectural solution to the scalability problem.
